# Predictive value of multimodal models combining CT body composition and clinical variables for short-term postoperative complications in colorectal cancer: a systematic review and meta-analysis

**DOI:** 10.3389/fnut.2026.1880518

**Published:** 2026-10-06

**Authors:** Yiheng Li, Chi Xue, Xinwei Zhang, Zhi Zhu

**Affiliations:** Department of Surgical Oncology, The First Affiliated Hospital of China Medical University, Shenyang, China

**Keywords:** body composition, colorectal cancer, machine learning, meta-analysis, precision nutrition, sarcopenic obesity

## Abstract

**Background:**

Malnutrition and sarcopenic obesity are prevalent in colorectal cancer (CRC) and linked to adverse surgical outcomes. Conventional screening often overlooks this occult malnutrition. Preoperative computed tomography (CT)-based body composition analysis (BCA) provides objective biomarkers for muscle depletion and fat distribution. This systematic review evaluates multimodal machine learning (ML) models integrating BCA and clinical variables to predict CRC postoperative complications and inform precision nutrition strategies.

**Methods:**

We systematically searched PubMed, Embase, Web of Science, and Cochrane Library (inception to April 13, 2026) for ML models predicting postoperative complications in CRC using BCA. Quality was assessed using PROBAST. Pooled area under the curve (AUC) was estimated via a random-effects model, alongside subgroup analyses.

**Results:**

Eight studies (*n* = 7,835) were included. The pooled AUC was 0.84 (95% CI: 0.76-0.89), indicating good discriminative performance, although substantial heterogeneity was present (*I*^2^ = 79.7%). Nomogram-based models showed a pooled AUC of 0.87 (95% CI: 0.78-0.92). For anastomotic leakage, the pooled AUC was 0.83 (95% CI: 0.35-0.98), although this estimate was imprecise because only two studies contributed to this subgroup.

**Conclusion:**

Multimodal ML models integrating BCA biomarkers offer a robust, data-driven framework for preoperative risk stratification in CRC. Identifying high-risk body composition phenotypes may facilitate tailored prehabilitation and targeted immunonutrition strategies. Future efforts must prioritize standardized BCA and multi-center external validation.

**Systematic review registration:**

CRD420261364363.

## 1 Introduction

Colorectal cancer (CRC) remains one of the most prevalent malignancies globally, with surgical resection serving as the cornerstone of curative-intent treatment (1). Despite substantial advances in minimally invasive surgery, robotic-assisted platforms, and the implementation of Enhanced Recovery After Surgery (ERAS) protocols, the overall postoperative complication rate after colorectal cancer (CRC) resection remains persistently elevated—ranging from 20 to 30%. Of particular clinical concern are severe complications including anastomotic leakage (AL) and surgical site infection (SSI), which continue to represent major contributors to morbidity, prolonged hospitalization, and treatment failure (2). Severe postoperative complications not only significantly prolong hospital length of stay and substantially increase healthcare expenditures, but also frequently delay the initiation of adjuvant chemotherapy—thereby compromising long-term oncologic outcomes, including disease-free survival (DFS) and overall survival (OS) (3). Therefore, the development of preoperatively applicable risk stratification tools—capable of accurately identifying high-risk patients and informing evidence-based clinical decisions—is critically needed to optimize perioperative outcomes in colorectal cancer surgery.

Historically, clinicians have relied on established risk assessment tools—including the American Society of Anesthesiologists (ASA) Physical Status Classification and the POSSUM (Physiological and Operative Severity Score for the Enumeration of Mortality and Morbidity) system—yet these conventional models are limited by their reliance on predefined variables and linear assumptions and a sparse set of routinely available clinical variables. Consequently, they lack the capacity to model the complex, nonlinear interplay among demographic, physiological, procedural, and disease-specific factors that determine perioperative risk in contemporary colorectal surgery. Robust evidence from recent multicenter cohort studies demonstrates consistently suboptimal discrimination for major postoperative complications (e.g., anastomotic leakage, intra-abdominal sepsis), with AUC values clustering tightly around 0.65 (range: 0.62–0.67)—well below the minimum threshold of 0.75 required for clinically actionable risk stratification. This persistent shortfall underscores a critical gap: existing tools cannot reliably guide precision-oriented, patient-tailored perioperative management (4, 5).

In recent years, objective body composition analysis (BCA) based on preoperative computed tomography (CT) images has become an important frontier direction in surgical oncology research. Accumulating clinical evidence indicates that sarcopenia status quantified by skeletal muscle index (SMI) and central obesity characterized by visceral fat area (VFA) significantly affect the postoperative complication rate, risk of prolonged hospital stay, and long-term survival prognosis of colorectal cancer (CRC) patients through multiple biological pathways, including regulating systemic inflammatory response, immunosuppressive microenvironment, and metabolic disorders (6). However, reliance solely on imaging-derived body composition parameters fails to capture the integrated impact of patient-level comorbidities (e.g., diabetes mellitus, chronic kidney disease) and tumor-intrinsic biological features (e.g., microsatellite instability status, RAS/BRAF mutation profile, and density of CD8+ tumor-infiltrating lymphocytes), which may limit predictive performance, particularly in external validation settings, and reduce the clinical applicability of single-modality models.

To overcome the inherent limitations of single-dimensional prediction, researchers have proposed a structured integration of high-dimensional body composition imaging features—including skeletal muscle radiodensity (Hounsfield units), intramuscular fat infiltration (quantified as muscle fat fraction), and visceral-to-subcutaneous adipose tissue ratio (VAT/SAT)—with multidimensional clinical–pathological variables such as ASA physical status classification, preoperative serum albumin concentration, tumor TNM stage, number of harvested lymph nodes, and receipt of adjuvant chemotherapy or radiotherapy. This integration forms the foundation of a multimodal data fusion–driven patient digital phenotype model. Given the high dimensionality, heterogeneity, and pervasive nonlinear interactions within these multimodal datasets, conventional approaches may have limited capacity to capture complex nonlinear interactions among high-dimensional multimodal predictors. Consequently, the field of medical artificial intelligence is increasingly adopting rigorously validated, interpretable, and robust machine learning algorithms—including calibration-optimized nomograms, ensemble-based random forests, and gradient-boosted decision trees (XGBoost)—to uncover synergistic predictive patterns across imaging, clinical, and pathological domains, thereby enhancing both discrimination accuracy (e.g., AUC > 0.85) and clinical utility for predicting severe postoperative complications, including anastomotic leakage, sepsis, and unplanned 30-day hospital readmission.

Although machine learning models based on multimodal data fusion have shown significant potential in predicting postoperative complications of colorectal cancer, a unified methodological consensus has not yet been reached in this field. Different independent cohort studies have high heterogeneity in core modeling steps, including algorithm architecture selection (such as whether to include deep learning modules), feature engineering strategies (such as whether to extract image features through texture analysis or automatic extraction by deep learning), and target endpoint definitions (such as modeling anastomotic leakage (AL) and surgical site infection (SSI) separately or combining them into a “infectious/structural complications” composite endpoint). In addition, most models are limited by single-center retrospective design, small sample size (median *n* < 300), and low positive event rate (AL incidence is often 5–12%). Although they perform well in internal validation, they show typical overfitting signals such as AUC reduction ≥0.10 and calibration slope deviation from 1.0 by more than ±0.2 in external prospective cohorts, which seriously restricts their clinical translation efficacy in the real world (7). To date, no high-quality systematic review and meta-analysis—adhering to PRISMA 2020 guidelines, pre-registered in PROSPERO, and incorporating rigorous risk-of-bias assessment using PROBAST—has been conducted to quantitatively synthesize the discriminative performance, primarily pooled AUC/C-statistic, of multimodal prediction models integrating CT-derived body-composition features with clinical variables for short-term postoperative complications following colorectal cancer surgery.

Therefore, this study rigorously follows the PRISMA 2020 statement and conducts a pre-registered (PROSPERO CRD420261364363), protocol-driven meta-analysis to quantitatively synthesize the discriminative performance, primarily pooled AUC/C-statistic, of multimodal prediction models integrating CT-derived body-composition features with clinical variables for short-term postoperative complications following colorectal cancer surgery. Furthermore, all included studies underwent critical appraisal using PROBAST (Prediction Model Risk of Bias Assessment Tool) to assess risk of bias across four domains: participants, predictors, outcome, and analysis (8). Through subgroup analyses, potential sources of heterogeneity, including variation by model derivation methodology/algorithmic paradigm and target postoperative complication phenotype, were explored. Analyses beyond the registered model-derivation stratification were interpreted as exploratory.

## 2 Methods

### 2.1 Literature search and study selection criteria (PICOS framework)

This systematic review and meta-analysis was conducted in accordance with the PRISMA 2020 statement (9). Eligibility criteria were prospectively defined using the PICOS framework:

Population: Adults (≥18 years) with colorectal cancer who underwent surgical resection.

Intervention/Model: Multivariable prediction models integrating at least one CT-derived body-composition metric (e.g., SMI, VFA, or PMI) with clinical, nutritional, inflammatory, or other routinely available patient-level variables. The registered protocol referred primarily to clinical nomograms; eligibility was operationalized more broadly to include functionally equivalent multivariable prediction models regardless of model form.

Comparator: No comparator was required for study eligibility. The PROSPERO registration explicitly states that the review does not have comparators.

Outcome: Quantitative measures of predictive discrimination, primarily AUC/C-statistic, with associated 95% confidence intervals (CIs) or standard errors (SEs), reported for short-term postoperative complications following colorectal cancer surgery. Eligible outcomes included overall or composite postoperative complications and specific postoperative complications, such as anastomotic leakage (AL), prolonged postoperative ileus (PPOI), parastomal hernia, intra-abdominal infection, and surgical site infection (SSI). Complication-specific subgroup analyses were performed only when sufficient studies were available.

Study Design: Primary studies reporting original data from retrospective or prospective observational cohort designs, with clearly documented participant enrollment, predictor measurement, outcome adjudication, and model development/validation procedures.

### 2.2 Risk of Bias and methodological quality assessment

Two independent reviewers critically appraised the methodological quality of all included studies using the updated PROBAST (Prediction Model Risk Of Bias ASsessment Tool) (10). PROBAST evaluates risk of bias across four prespecified domains: participants, predictors, outcome, and analysis (D4). For each domain, judgments were made according to standardized signaling questions—e.g., whether participant selection introduced spectrum bias; whether predictor measurement was standardized and blinded; whether outcome adjudication was objective and independent; and whether model development included appropriate handling of overfitting (e.g., via bootstrapping or penalization), rigorous internal validation (e.g., cross-validation or split-sample), and transparent reporting of calibration and discrimination metrics. A study was classified as having “high overall risk of bias” only if it was rated as high risk in ≥1 domain *and* that domain deficiency directly threatened the validity of the model’s predictive performance estimates (e.g., absence of external validation for a model claiming generalizability; use of univariable selection without adjustment for multiple testing). Disagreements were resolved through consensus or arbitration by a third reviewer. Results were visualized using traffic-light plots and stacked bar charts.

### 2.3 Data extraction and statistical analysis

All statistical analyses were performed using R software (version 4.5.3) with the meta package (11).

Effect Size Synthesis and Heterogeneity Assessment: For each included study, the area under the receiver operating characteristic curve (AUC) and its corresponding standard error (SE) were extracted as the primary effect size. Given anticipated clinical and methodological heterogeneity across studies—including variations in patient demographics, imaging acquisition protocols, body composition feature extraction methods, and surgical management pathways—a random-effects model (DerSimonian–Laird estimator, inverse-variance weighting) was employed for pooled effect estimation. Heterogeneity was quantified using Cochran’s *Q* test (with significance set at *p* < 0.10) and the *I*^2^ statistic; *I*^2^ > 50% was interpreted as indicating substantial heterogeneity.

Subgroup analyses were conducted to investigate potential sources of heterogeneity and model generalizability. Analyses considered model derivation methodology/algorithmic paradigm and target postoperative complication phenotype. Analyses beyond the registered model-derivation stratification were interpreted as exploratory. To ensure stable between-subgroup variance estimation, particularly given the limited number of studies per subgroup, a common *τ*^2^ estimator was applied across subgroups, and the Knapp–Hartung adjustment was used to derive 95% confidence intervals (12).

Sensitivity Analysis and Assessment of Small-Study Effects: A leave-one-out sensitivity analysis was performed to assess the influence of individual studies on the pooled AUC estimate and overall heterogeneity (*I*^2^ and *τ*^2^). Small-study effects—including potential publication bias—were evaluated through visual inspection of funnel plots and formal testing using Egger’s linear regression test (*p* < 0.05 considered statistically significant) (13).

## 3 Results

### 3.1 Study characteristics and risk of bias assessment

This meta-analysis included eight independent cohort studies (Figure 1, Table 1) (14–20, 42). As assessed by the PROBAST tool (Figures 2, 3), methodological rigor was consistently high across clinical domains: 100% of studies were rated at low risk of bias in the Outcome domain (D3); 87.5% (7/8) achieved low risk in both the Participants (D1) and Predictors (D2) domains. In contrast, only 50% (4/8) were rated at low risk in the Analysis domain (D4), while 37.5% (3/8) were classified as high risk—primarily due to insufficient events-per-variable (EPV < 10) in high-dimensional multimodal modeling and absence of external validation in independent cohorts. Consequently, four studies were deemed to have high overall risk of bias, underscoring a persistent methodological gap in current AI-based prediction model development for colorectal surgery.

**Figure 1:**
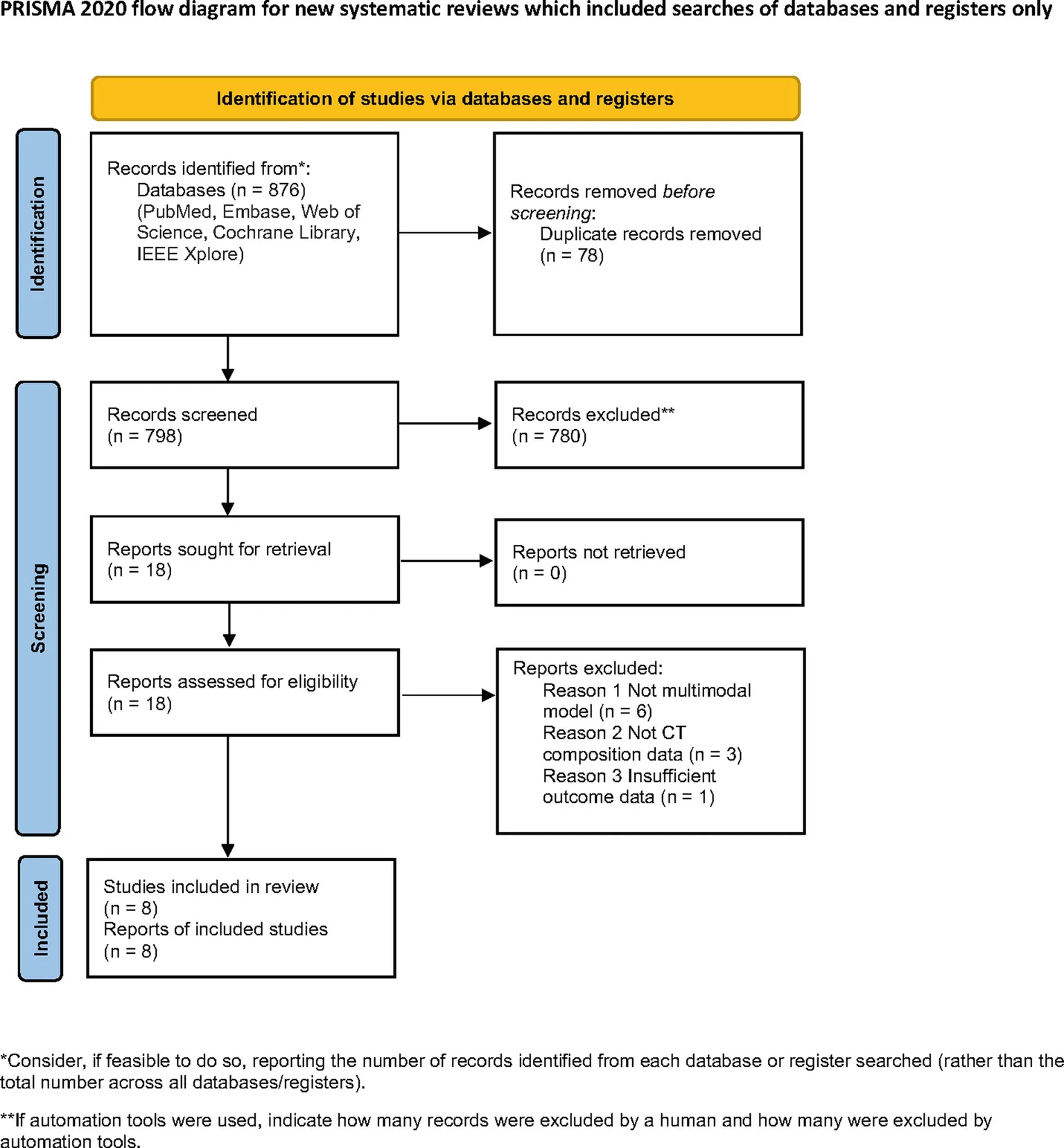
PRISMA flow diagram of the study selection process.

**Table 1:** Characteristics of the included studies and multimodal prediction models.

Study	Year	Cancer_Type	Total_N	Outcome_Definition	CT_Predictor	Clinical_Predictor	Model_Type	AUC_Multi	Lower_CI	Upper_CI	SE_Multi
Xiu_2024 (20)	2024	Colorectal	2368	PPOI	Sarcopenia (SMI)	Age, Sex, PNI, CCI, Fluid	Nomogram	0.838	0.738	0.911	0.04413
van Kooten_2023 (18)	2023	Rectosigmoid	146	Anastomotic leakage	PMI / SMI	Age, Sex, Comorbidity, CRT	Logistic Regression	0.798	0.71	0.99	0.071428571
Fu_2026 (42)	2026	Colostomy pts	430	Parastomal hernia	Various CT indices	elderly patients (age ≥ 65 years), Female, body mass index, visceral fat area (VFA) subcutaneous fat area (SFA), surrounding parastomal fat tissue (SPFT), maximum abdominal wall defect diameter	Prognostic model	0.867	0.804	0.933	0.032908163
Lu_2026 (14)	2026	Colon	475	Postoperative complications	SMI	Albumin	Logistic Regression	0.658	0.59	0.726	0.034693878
Ma_2023 (15)	2023	Colon (Stage II-III)	248	Postoperative complications	SMI, VFI	MLR, SII, NRS	Nomogram	0.901	0.816	0.986	0.043367347
Pei_2022 (16)	2022	Colorectal	402	Intra-abdominal infection	Muscle & Subcutaneous Fat	Albumin, LWR <0.17	Nomogram	0.931	0.815	0.971	0.039795918
Tan_2023 (17)	2023	Colon	85	Postoperative complications	VFA,SFA,MonoE,IoD,Z-eff	Gender,Age,Amenia,TNMstage,HER-2,Ki-67,EGFR	Stepwise Regression	0.84	0.74	0.91	0.043367347
Xiang_2022 (19)	2022	Colorectal	3681	Anastomotic leakage	SMA,VFA	gender, blood glucose, NRS	Nomogram	0.848	0.781	0.914	0.033928571

**Figure 2:**
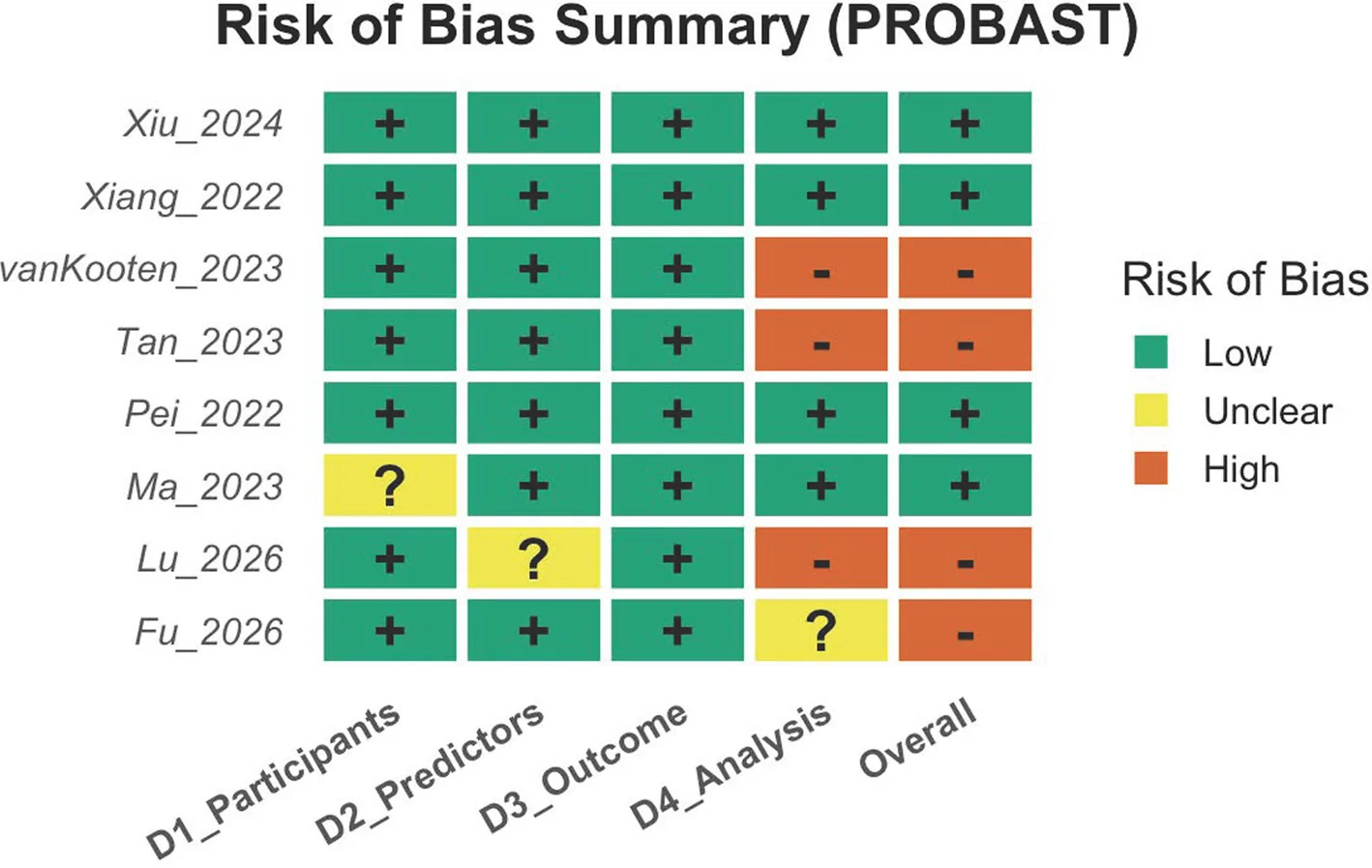
PROBAST risk of bias summary (traffic-light plot). Reviewers’ judgments about each risk of bias domain (Participants, Predictors, Outcome, and Analysis) for each included study evaluating the multimodal prediction models.

**Figure 3:**
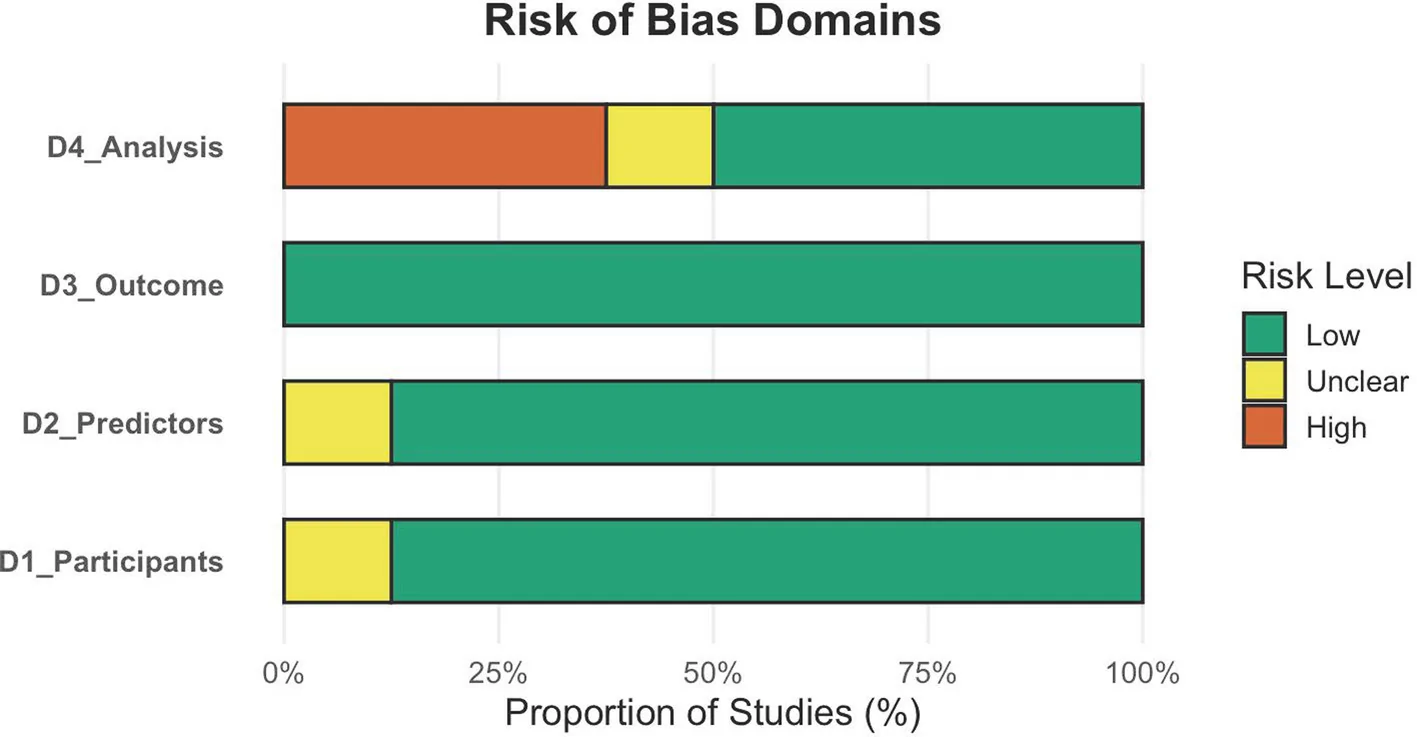
PROBAST risk of bias graph (stacked bar chart). Proportion of studies with low, high, or unclear risk of bias across the four PROBAST domains. Note the high risk of bias concentrated in the Analysis domain (D4) due to inadequate events-per-variable (EPV) and lack of external validation.

### 3.2 Overall predictive performance of multimodal models

Figure 4 presents the forest plot of pooled AUC estimates for multimodal machine learning models predicting postoperative complications in patients with colorectal cancer (CRC). Under the random-effects model, the summary AUC across all eight studies was 0.84 (95% CI: 0.76–0.89). Substantial heterogeneity was observed (*I*^2^ = 79.7%, *p* < 0.0001), indicating good-to-excellent discriminative ability. This robust performance substantiates the clinical value of integrating quantitative body composition features from preoperative imaging with multidimensional clinical–pathological variables. AUC = 0.84 (95% CI: 0.76–0.89), *I*^2^ = 79.7%, *p* < 0.0001.

**Figure 4:**
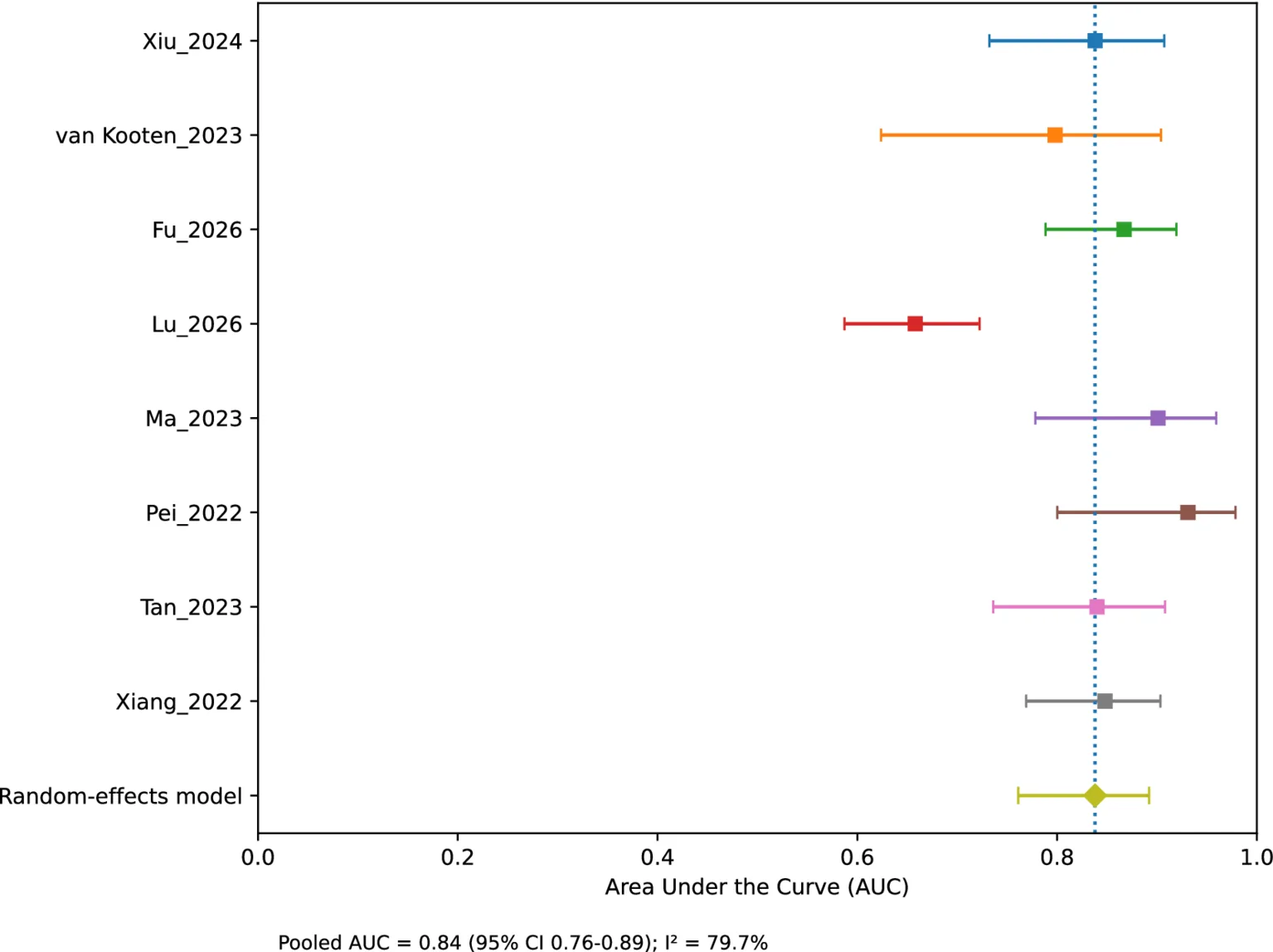
Forest plot of the pooled area under the curve (AUC) for multimodal machine learning models. The plot illustrates the discriminative performance of the models in predicting overall postoperative complications in patients undergoing colorectal cancer surgery. The summary AUC across all eight included studies was 0.84 (95% CI: 0.76–0.89) based on a random-effects model.

### 3.3 Subgroup analyses to explore heterogeneity and generalizability

Subgroup analyses by model derivation methodology/algorithmic paradigm and target complication phenotype; analyses beyond registered model-derivation stratification are exploratory; common *τ*^2^ and Knapp-Hartung adjustment were used.

By algorithmic paradigm (Figure 5): Exploratory subgroup analysis suggested variation in predictive performance by model type. Nomogram-based models showed a pooled AUC of 0.87 (95% CI: 0.78–0.92; *I*^2^ = 0%), whereas logistic regression models showed a pooled AUC of 0.69 (95% CI: 0.05–0.99; *I*^2^ = 57.5%). Given the small number of studies and the wide confidence interval for logistic regression, this comparison should be interpreted as exploratory.

**Figure 5:**
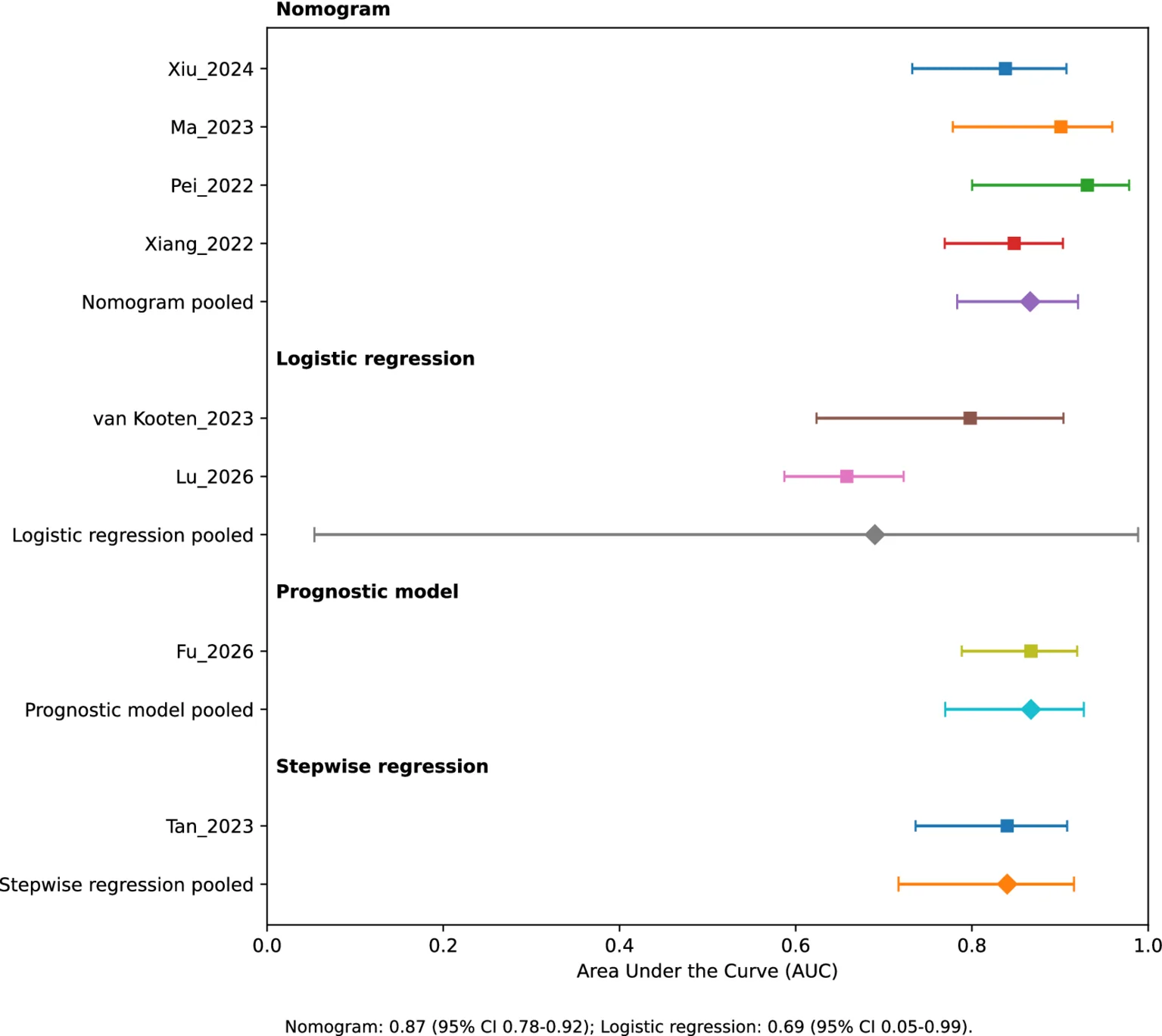
Subgroup analysis of predictive performance stratified by algorithmic paradigm. Nomogram-based models showed a pooled AUC of 0.87 (95% CI: 0.78–0.92; *I*^2^ = 0%), whereas logistic regression models showed a pooled AUC of 0.69 (95% CI: 0.05–0.99; *I*^2^ = 57.5%). These subgroup comparisons should be interpreted as exploratory.

By target complication (Figure 6): Exploratory subgroup analyses showed broadly consistent discrimination across the evaluated postoperative complication phenotypes. For anastomotic leakage (AL), the pooled AUC was 0.83 (95% CI: 0.35–0.98). For postoperative complications, the pooled AUC was 0.80 (95% CI: 0.36–0.97). Complication-specific estimates should be interpreted cautiously because only a small number of studies contributed to individual outcome categories.

**Figure 6:**
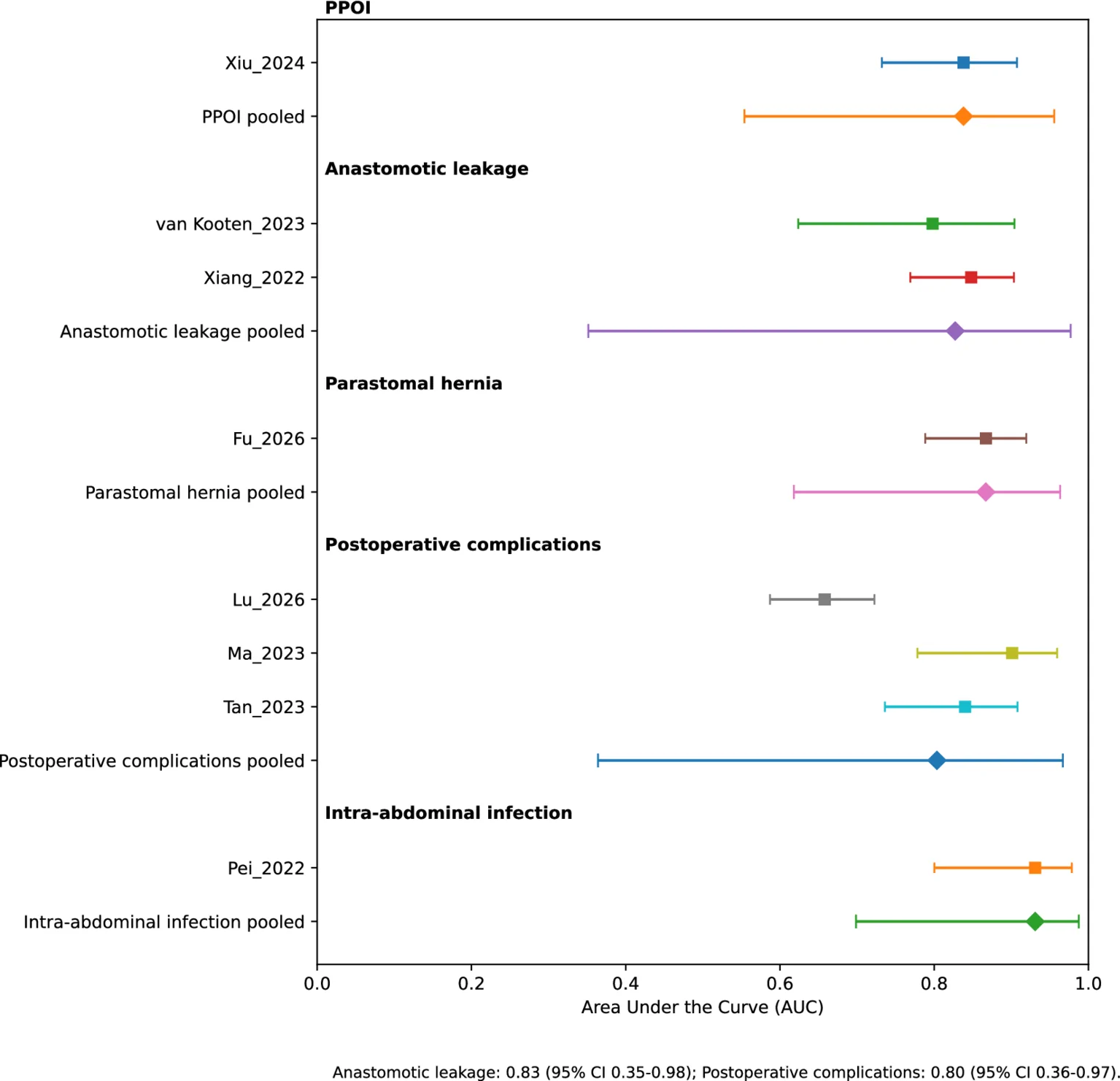
Subgroup analysis of predictive performance stratified by target complication phenotype. The pooled AUC was 0.83 (95% *CI*: 0.35–0.98) for anastomotic leakage and 0.80 (95% *CI*: 0.36–0.97) for postoperative complications. Estimates for individual complication categories should be interpreted cautiously because of the limited number of contributing studies.

### 3.4 Sensitivity analysis

Leave-one-out sensitivity analysis (Figure 7) demonstrated that the pooled AUC remained broadly stable after exclusion of individual studies. Excluding Lu et al. (14) increased the pooled AUC to 0.86 (95% CI: 0.82–0.88) and reduced heterogeneity to *I*^2^ = 0%, identifying this study as the primary contributor to between-study heterogeneity.

**Figure 7:**
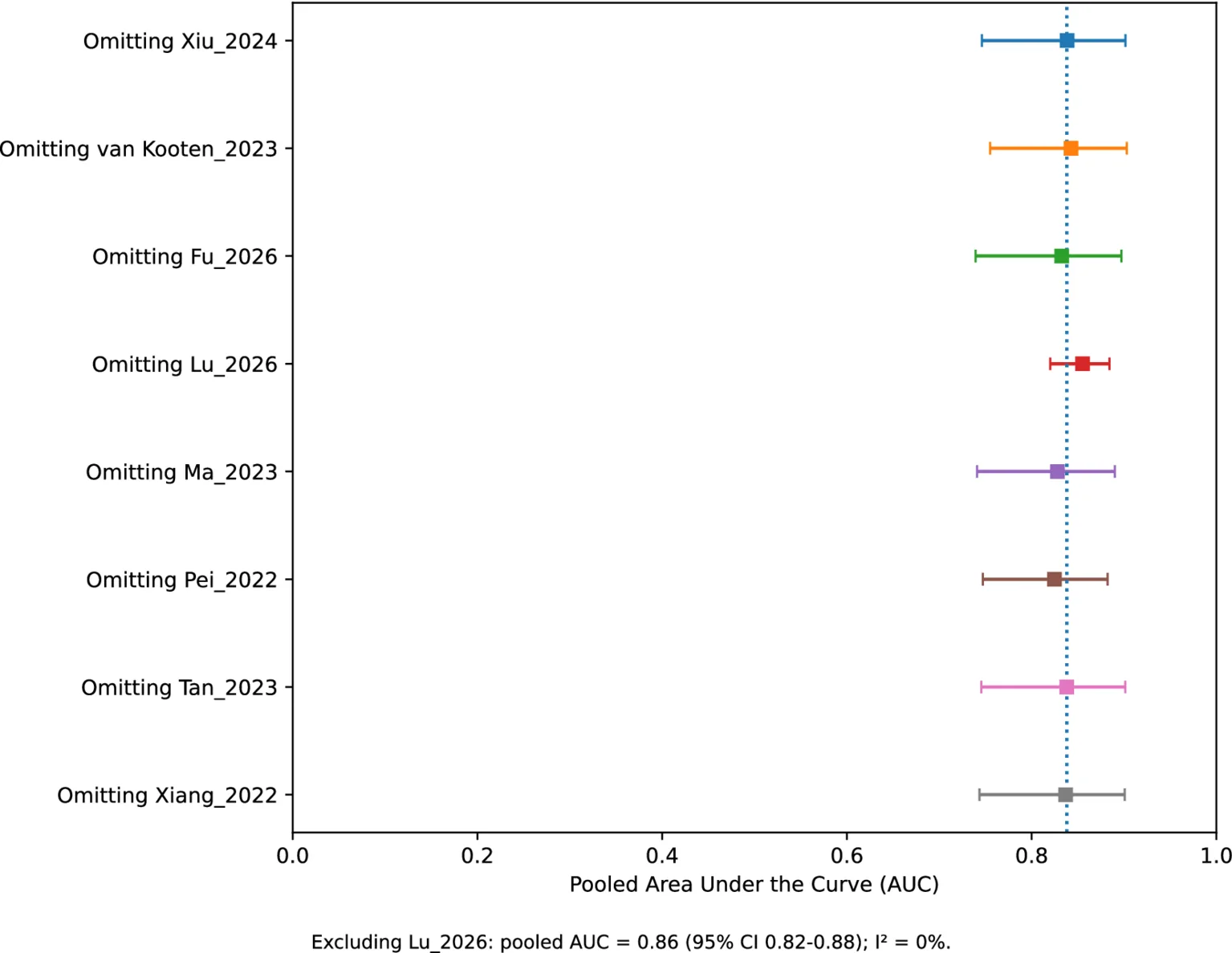
Leave-one-out sensitivity analysis. The plot visualizes the fluctuation of the summary AUC and 95% CI when each primary study is sequentially excluded from the meta-analysis, confirming the overall robustness of the pooled estimate and identifying the primary outlier driving the statistical heterogeneity.

### 3.5 Assessment of small-study effects

Visual inspection of the funnel plot (Figure 8) suggested asymmetry. Egger’s regression test yielded an intercept of 4.42 (SE = 1.11, *t* = 3.99, p = 0.007), suggesting possible small-study effects. However, given the limited number of included studies (*k* = 8), the result is unstable and should not be interpreted as definitive evidence of publication bias.

**Figure 8:**
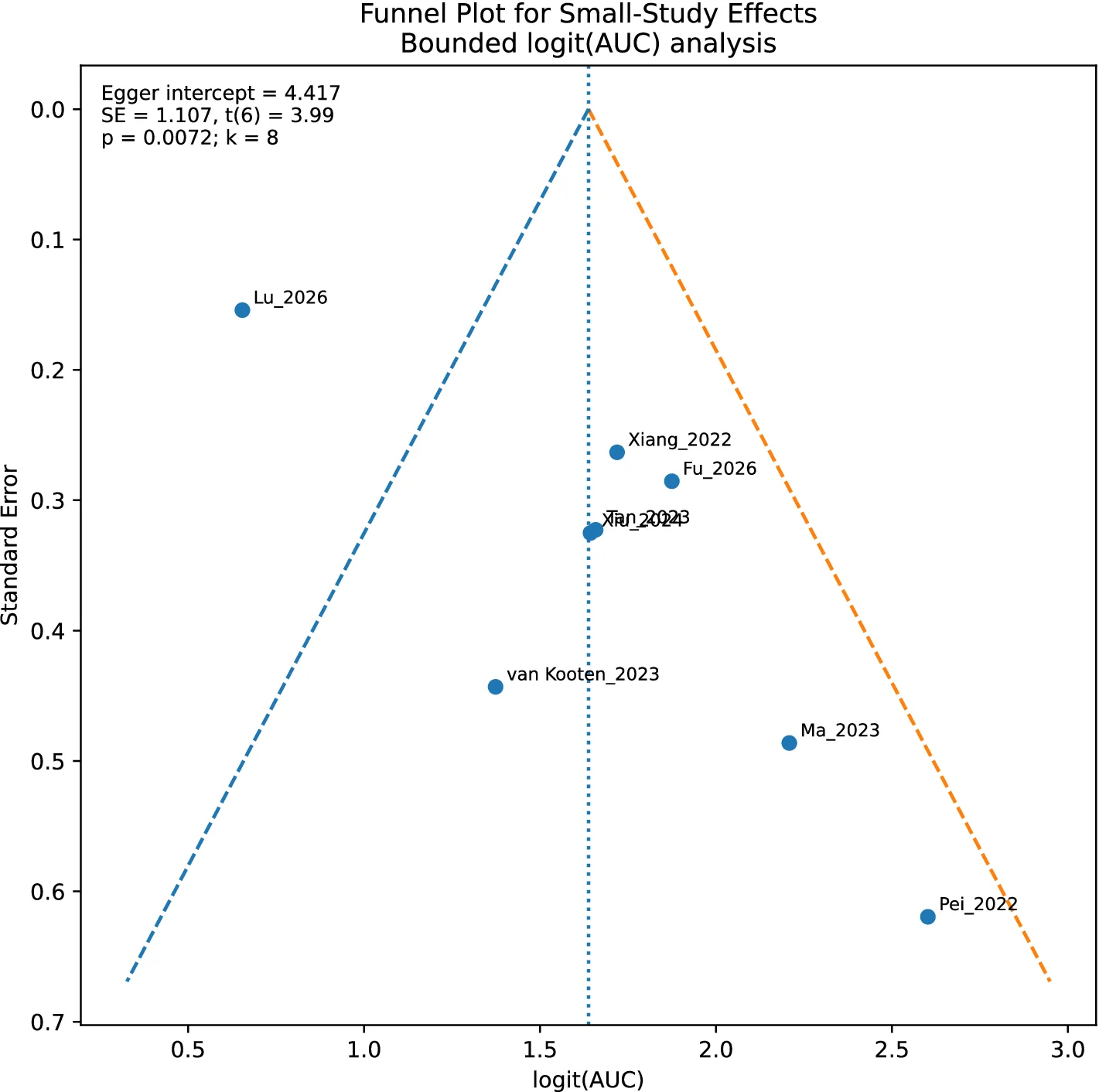
Funnel plot for the assessment of small-study effects using bounded logit(AUC) analysis. Visual inspection suggested asymmetry, and Egger’s regression test was statistically significant (intercept = 4.42, SE = 1.11, t = 3.99, *p* = 0.007). Given the small number of included studies (k = 8), this finding should be interpreted cautiously as possible small-study effects rather than definitive evidence of publication bias.

## 4 Discussion

### 4.1 Principal findings

To our knowledge, this is the first systematic review and meta-analysis to evaluate the predictive performance of multimodal machine learning models that integrate quantitative body composition features—derived from preoperative cross-sectional imaging—with multidimensional clinical–pathological variables for postoperative complication risk stratification in patients undergoing colorectal cancer (CRC) surgery. The pooled analysis showed good discriminative performance (AUC = 0.84). Exploratory subgroup analyses suggested broadly consistent performance across the evaluated postoperative complication phenotypes, including anastomotic leakage (AL; pooled AUC = 0.83), although complication-specific estimates were imprecise because of the limited number of contributing studies. These findings suggest potential applicability across postoperative complication phenotypes but require further prospective and external validation.

### 4.2 Clinical and biological plausibility of multimodal integration

The broadly consistent predictive performance observed across the evaluated postoperative complication phenotypes supports the biological plausibility of multimodal integration, although complication-specific estimates remain limited by the small number of studies within individual outcome categories. Conventional risk scores based on BMI or ASA classification frequently overlook sarcopenic obesity—a high-risk phenotype defined by concurrent depletion of skeletal muscle mass and accumulation of visceral adipose tissue (21). In contrast, multimodal models incorporate objective, quantitative imaging biomarkers—including skeletal muscle index (SMI), visceral adipose tissue area (VAT), and their ratio (VAT/SMI)—that serve as surrogates for systemic inflammation, insulin resistance, mitochondrial dysfunction, and immunosenescence (22, 23). These anatomically anchored features interact nonlinearly with clinical–pathological variables (e.g., tumor stage, comorbidities, neoadjuvant treatment), generating pathophysiologically interpretable signals for impaired collagen synthesis, microvascular insufficiency, and delayed epithelialization (23). Such mechanistic alignment explains why multimodal integration demonstrated higher predictive performance than unidimensional clinical scoring systems in the included studies (e.g., POSSUM, ACS-NSQIP), which lack granularity to capture host–tumor–microenvironment interplay (24, 25).

### 4.3 Methodological insight: algorithm choice as a determinant of predictive ceiling

Exploratory subgroup analyses suggested variation in predictive performance by model type. Nomogram-based models showed a pooled AUC of 0.87 (95% CI: 0.78–0.92; *I*^2^ = 0%), whereas logistic regression models showed a pooled AUC of 0.69 (95% CI: 0.05–0.99; *I*^2^ = 57.5%). Lu_2026—a logistic-regression study rated high risk in PROBAST D4—was identified as an important contributor to overall heterogeneity. Given the small number of studies and the wide confidence interval for the logistic-regression subgroup, these findings should be interpreted as exploratory and hypothesis-generating rather than evidence of superiority.

### 4.4 Quality assessment and pathways forward: addressing the D4 gap

PROBAST assessment exposed a critical methodological gap: while studies demonstrated high fidelity in participant selection (D1), predictor measurement (D2), and outcome adjudication (D3), 37.5% (3/8) were rated as high risk in the Analysis domain (D4), while one additional study (12.5%) was rated as unclear risk. Three recurrent deficiencies emerged: (i) insufficient events-per-variable (EPV < 10) for rare complications such as AL, resulting in unstable coefficient estimation and overfitting during high-dimensional feature selection (26); (ii) reliance solely on single-center internal validation (e.g., k-fold cross-validation), producing optimistic and non-generalizable performance estimates (27); and (iii) inadequate handling of class imbalance (e.g., absence of SMOTE, ADASYN, or cost-sensitive learning) (28), compromising calibration, clinical net benefit, and decision curve validity. To bridge this gap, we recommend strict adherence to the TRIPOD+AI statement (29), prospective registration of analysis protocols, multicenter external validation in geographically and demographically diverse populations, and mandatory reporting of both discrimination (AUC/C-index) and calibration metrics (calibration slope, intercept, Brier score, and decision curve analysis) (45, 46).

### 4.5 Clinical implications and translational potential

This study demonstrates the tangible translational potential of multimodal prediction models in bridging the gap between data science innovation and real-world surgical decision-making. Unlike conventional tools used solely for *post hoc* audit or population-level risk scoring, these models may provide a potential framework for prospective, individualized, and biologically informed clinical support across the entire perioperative continuum. Their value manifests across four interlinked domains:

1. *Enabling precision prehabilitation and targeted nutrition*: By integrating objective, quantitative body composition metrics—such as skeletal muscle index (SMI) and visceral adipose tissue area (VAT)—the model transforms probabilistic risk into actionable pathophysiological insight. For patients flagged as high-risk for anastomotic leakage (AL) or surgical site infection (SSI) due to sarcopenia or sarcopenic obesity, a structured 2–4-week prehabilitation program may be considered *before* surgery: supervised resistance training, protein- and immunonutrient-enriched dietary support (e.g., arginine, omega-3 fatty acids, vitamin D) (30), and targeted physical therapy. Such interventions are evidence-based to improve muscle mass, reduce systemic inflammation, and enhance immune competence—thereby modifying the host microenvironment to favor optimal wound healing (31).
2. *Informing individualized surgical decision-making*: Validated models may eventually complement multidisciplinary surgical decision-making, but their use to select specific operative strategies requires prospective impact studies.
3. *Optimizing perioperative resource allocation within ERAS frameworks*: Within Enhanced Recovery After Surgery (ERAS) protocols (33), the model serves as a dynamic triage tool. Low-risk patients may safely undergo accelerated discharge pathways—including same-day discharge after laparoscopic surgery or early transition to outpatient rehabilitation—while high-risk patients benefit from proactive resource allocation: reserved ICU or step-down unit beds, scheduled early postoperative CRP and procalcitonin monitoring (34), and timely activation of rapid response teams. This risk-stratified approach improves patient safety without compromising efficiency—and has demonstrated cost-effectiveness in recent health economic modeling studies (e.g., incremental cost-effectiveness ratio <$50,000/QALY) (35).
4. *Future direction*: Toward Digital Health Integration: As deep learning–based segmentation algorithms continue to mature toward clinical-grade robustness, automated body composition analysis may eventually become integrated into routine radiology workflows, pending appropriate validation and regulatory review (36). Upon completion of standard preoperative CT, picture archiving and communication system (PACS)-integrated AI modules may automatically quantify SMI, VAT, SAT, and their ratios; these values could be integrated into the electronic health record (EHR) and merged with structured clinical data to support risk assessment. Clinicians receive actionable alerts—not raw algorithm outputs—minimizing cognitive load and enabling “zero-friction” AI-assisted decision support at the point of care (37).

### 4.6 Limitations

While this meta-analysis yields findings with substantial clinical and translational relevance, several important limitations warrant careful consideration:

First, constraints in the number of eligible studies and statistical power: True multimodal models integrating quantitative body composition features from preoperative imaging with multidimensional clinical–pathological variables remain nascent in colorectal surgery. Consequently, only eight independent cohorts met our stringent PICOS eligibility criteria. This limited pool constrains the statistical power of tests for small-study effects (e.g., Egger’s test) (38) and precludes more granular analyses—such as meta-regression—to explore the influence of clinically relevant moderators (e.g., tumor stage [Stage I–II vs. III–IV], surgical approach [laparoscopic vs. robotic-assisted], or neoadjuvant therapy exposure).

Second, inherent biases of retrospective study designs: The vast majority of included primary studies employed retrospective cohort designs. These are susceptible to selection bias (e.g., exclusion of patients with incomplete imaging or missing follow-up), information bias (e.g., inconsistent complication documentation across chart reviewers), and residual confounding—even after multivariable adjustment. Critically, retrospective data cannot capture dynamic changes in body composition induced by prehabilitation interventions (e.g., resistance training or nutritional supplementation), meaning current models reflect static baseline risk rather than modifiable physiological trajectories. Prospective, multicenter randomized controlled trials (RCTs) are therefore essential to validate whether model-informed risk stratification meaningfully improves clinical outcomes.

Third, methodological heterogeneity in feature extraction and outcome definition: Substantial variability persists across studies in technical implementation. In body composition quantification: segmentation tools differ (e.g., SliceOmatic, ImageJ, or deep learning–based platforms); Hounsfield unit (HU) thresholds for skeletal muscle (−29 to −50 HU) and adipose tissue (−190 to −30 HU) are inconsistently applied; and anatomical levels for CT slice selection vary (e.g., third lumbar vertebra [L3] vs. umbilical level) (39). In clinical outcome adjudication: definitions of target complications lack harmonization—e.g., surgical site infection (SSI) is variously defined per CDC criteria, Clavien–Dindo grade ≥II, or grade ≥III; anastomotic leakage (AL) is reported using diverse radiologic or clinical criteria (40). These nonstandardized technical and clinical practices contribute meaningfully to residual heterogeneity and limit direct comparability across models.

Fourth, absence of molecular and genomic dimensions: Current multimodal frameworks remain anchored in macroscopic anatomy (body composition) and standard clinical phenotypes, largely omitting critical molecular biomarkers that profoundly influence the tumor microenvironment and tissue healing. Emerging evidence indicates that factors such as microsatellite instability (MSI), KRAS/BRAF mutational status, and circulating tumor DNA (ctDNA) dynamics are intimately linked to host systemic inflammation and postoperative outcomes. Evolving these models into true “radiogenomic” (imaging-genomic) or pan-omic predictive systems represents a crucial frontier for bridging the macroscopic-microscopic divide in future surgical AI development (41).

## 5 Conclusion

This systematic review and meta-analysis suggests that multimodal prediction models integrating quantitative body-composition features from preoperative imaging with multidimensional clinical–pathological variables demonstrate promising discrimination for postoperative complications following colorectal cancer surgery. These models combine objective imaging-derived body-composition markers with clinical variables and showed promising discrimination; whether they outperform conventional risk scores requires prospective head-to-head validation. Promising discrimination was observed across several clinically relevant complication phenotypes; however, the limited number of studies per outcome category precludes definitive conclusions regarding complication-specific performance.

Exploratory subgroup analyses suggested variation by model type, but these findings are hypothesis-generating and require confirmation in larger head-to-head validation studies. Methodological rigor—particularly appropriate control of overfitting, adequate events-per-variable, and external validation in independent cohorts—remains essential for assessing model reliability and clinical implementation readiness.

In summary, multimodal machine learning models may represent a promising framework supporting precision prehabilitation and physiology-guided surgical decision-making in colorectal surgery. To bridge the translational “last mile” from algorithm development to point-of-care integration, three coordinated priorities are essential. First, international consensus is needed on standardized, automated body composition quantification—including L3-level CT segmentation with harmonized Hounsfield unit thresholds and unified reporting templates. Second, prospective multicenter external validation studies should adhere strictly to the TRIPOD+AI statement, with mandatory reporting of calibration metrics, decision curve analysis, and clinical net benefit. Third, future models should expand toward radiogenomic–clinical tri-modal architectures that integrate tumor molecular profiles—including microsatellite instability status, circulating tumor DNA burden, and KRAS/BRAF mutational status—with imaging and clinical data to enhance both biological interpretability and predictive fidelity.

## Data Availability

The original contributions presented in the study are included in the article/Supplementary material, further inquiries can be directed to the corresponding author.
