# Supplementary material for: Predictive value of multimodal models combining CT body composition and clinical variables for short-term postoperative complications in colorectal cancer: a systematic review and meta-analysis
Source: Front Nutr. 2026 Oct 6;13:1880518. doi: 10.3389/fnut.2026.1880518 (PMC13680152; doi:10.3389/fnut.2026.1880518)
Supplement: Supplementary file 1 [file Supplementary_file_1.docx]

| **Database** | **Search Strategy** | **Filters / Limits** | **Date of Search** |
| --- | --- | --- | --- |
| **PubMed** | ("Colorectal Neoplasms"[Mesh] OR "colorectal cancer"[tiab] OR "colon cancer"[tiab] OR "rectal cancer"[tiab] OR "colorectal tumor*"[tiab] OR "colorectal neoplasm*"[tiab] OR "colorectal carcinoma*"[tiab] OR CRC[tiab]) AND ("Body Composition"[Mesh] OR "Sarcopenia"[Mesh] OR "Intra-Abdominal Fat"[Mesh] OR "body composition"[tiab] OR sarcopeni*[tiab] OR "visceral fat"[tiab] OR "visceral adiposity"[tiab] OR "skeletal muscle"[tiab] OR radiomic*[tiab] OR "computed tomography"[tiab] OR "CT image*"[tiab]) AND ("Machine Learning"[Mesh] OR "Artificial Intelligence"[Mesh] OR "Deep Learning"[Mesh] OR "Models, Statistical"[Mesh] OR "machine learning"[tiab] OR "artificial intelligence"[tiab] OR "deep learning"[tiab] OR "neural network*"[tiab] OR "predictive model*"[tiab] OR "prediction model*"[tiab] OR algorithm*[tiab] OR nomogram*[tiab] OR xgboost[tiab] OR "random forest"[tiab]) | None | 2026-04-13 |
| **Web of Science** | TS=("colorectal cancer" OR "colon cancer" OR "rectal cancer" OR "colorectal tumor*" OR "colorectal carcinoma*" OR CRC) AND TS=("body composition" OR sarcopeni* OR "visceral fat" OR "visceral adiposity" OR "skeletal muscle" OR radiomic* OR "computed tomography" OR "CT image*") AND TS=("machine learning" OR "artificial intelligence" OR "deep learning" OR "neural network*" OR "predictive model*" OR algorithm* OR nomogram* OR xgboost OR "random forest") AND TS=("postoperative complication*" OR "surgical complication*" OR "anastomotic leak*" OR "surgical site infection*" OR morbidit*) | None | 2026-04-13 |
| **Embase** | ('colorectal tumor'/exp OR 'colorectal cancer':ab,ti OR 'colon cancer':ab,ti OR 'rectal cancer':ab,ti OR 'colorectal carcinoma*':ab,ti OR crc:ab,ti) AND ('body composition'/exp OR 'sarcopenia'/exp OR 'visceral fat'/exp OR 'body composition':ab,ti OR sarcopeni*:ab,ti OR 'visceral adiposity':ab,ti OR 'skeletal muscle':ab,ti OR radiomic*:ab,ti OR 'computed tomography':ab,ti) AND ('machine learning'/exp OR 'artificial intelligence'/exp OR 'deep learning'/exp OR 'machine learning':ab,ti OR 'deep learning':ab,ti OR 'neural network*':ab,ti OR 'predictive model*':ab,ti OR algorithm*:ab,ti OR nomogram*:ab,ti OR xgboost:ab,ti OR 'random forest':ab,ti) AND ('postoperative complication'/exp OR 'anastomotic leakage'/exp OR 'postoperative complication*':ab,ti OR 'surgical complication*':ab,ti OR 'anastomotic leak*':ab,ti OR morbidit*:ab,ti) | None | 2026-04-13 |
| **Cochrane Library** | ([mh "Colorectal Neoplasms"] OR "colorectal cancer":ti,ab,kw OR "colon cancer":ti,ab,kw OR "rectal cancer":ti,ab,kw OR CRC:ti,ab,kw) AND ([mh "Body Composition"] OR [mh Sarcopenia] OR [mh "Intra-Abdominal Fat"] OR "body composition":ti,ab,kw OR sarcopeni*:ti,ab,kw OR "visceral fat":ti,ab,kw OR radiomic*:ti,ab,kw OR "computed tomography":ti,ab,kw) AND ([mh "Machine Learning"] OR [mh "Artificial Intelligence"] OR [mh "Deep Learning"] OR "machine learning":ti,ab,kw OR "deep learning":ti,ab,kw OR "predictive model*":ti,ab,kw OR algorithm*:ti,ab,kw OR nomogram*:ti,ab,kw) AND ([mh "Postoperative Complications"] OR "postoperative complication*":ti,ab,kw OR "anastomotic leak*":ti,ab,kw OR morbidit*:ti,ab,kw) | None | 2026-04-13 |
| **IEEE Xplore** | ("Document Title":"colorectal cancer" OR "Abstract":"colorectal cancer" OR "Abstract":"rectal cancer" OR "Abstract":"colon cancer") AND ("Abstract":"body composition" OR "Abstract":"sarcopenia" OR "Abstract":"radiomics" OR "Abstract":"computed tomography") AND ("Abstract":"machine learning" OR "Abstract":"deep learning" OR "Abstract":"predictive model" OR "Abstract":"algorithm" OR "Abstract":"nomogram") AND ("Abstract":"complication" OR "Abstract":"anastomotic leak" OR "Abstract":"morbidity") | None | 2026-04-13 |
